# Acute phase proteins and white blood cell levels for prediction of infectious complications in status epilepticus

**DOI:** 10.1186/cc10555

**Published:** 2011-11-18

**Authors:** Raoul Sutter, Sarah Tschudin-Sutter, Leticia Grize, Andreas F Widmer, Stephan Marsch, Stephan Rüegg

**Affiliations:** 1 Division of Clinical Neurophysiology, Department of Neurology, University Hospital Basel, Peterplatz 1, Basel, 4003, Switzerland; 2 Intensive Care Unit, University Hospital Basel, Peterplatz 1, Basel, 4003, Switzerland; 3 Department of Infectious Diseases and Hospital Epidemiology, University Hospital Basel, Peterplatz 1, Basel, 4003 Switzerland; 4 Department of Epidemiology and Public Health, Swiss Tropical and Public Health Institute, Socinstrasse 57, Basel, 4002, Switzerland

## Abstract

**Introduction:**

Infections in status epilepticus (SE) patients result in severe morbidity making early diagnosis crucial. As SE may lead to inflammatory reaction, the value of acute phase proteins and white blood cells (WBC) for diagnosis of infections during SE may be important. We examined the reliability of C-reactive protein (CRP), procalcitonin (PCT), and WBC for diagnosis of infections during SE.

**Methods:**

All consecutive SE patients treated in the ICU from 2005 to 2009 were included. Clinical and microbiological records, and measurements of CRP and WBC during SE were analyzed. Subgroup analysis was performed for additional PCT measurements in the first 48 hours of SE.

**Results:**

A total of 22.5% of 160 consecutive SE patients had infections during SE. Single levels of CRP and WBC had no association with the presence of infections. Their linear changes over the first three days after SE onset were significantly associated with the presence of infections (*P *= 0.0012 for CRP, *P *= 0.0137 for WBC). Levels of PCT were available for 31 patients and did not differ significantly in patients with and without infections. Sensitivity of PCT and CRP was high (94% and 83%) and the negative predictive value of CRP increased over the first three days to 97%. Specificity was low, without improvement for different cut-offs.

**Conclusions:**

Single levels of CRP and WBC are not reliable for diagnosis of infections during SE, while their linear changes over time significantly correlate with the presence of infections. In addition, low levels of CRP and PCT rule out hospital-acquired infections in SE patients.

## Introduction

Infection rate of patients with status epilepticus (SE) is high and associated with increased morbidity, need for treatment escalation, prolonged hospital stay and additional resource utilization [1]. SE patients are at risk for ventilator-associated pneumonia (VAP) due to the need for mechanical ventilation during their state of altered consciousness. Therefore, early and accurate diagnosis of hospital-acquired infections during SE is crucial [1].

Since its identification in 1930, C-reactive protein (CRP) has been studied as a screening device for inflammation, a marker for disease activity, and as a diagnostic adjunct [2] as values of CRP may reflect the severity of inflammation or tissue injury [3,4]. Like many acute phase proteins, CRP is normally present in trace levels in serum but increases rapidly and dramatically in response to a variety of infectious or inflammatory conditions [5]. With the availability of rapid or bedside tests, determining its diagnostic value is of increasing importance [6].

Procalcitonin (PCT) is a pre-pro-peptide precursor of the thyroid hormone calcitonin. Circulating levels of the PCT can rise several thousand times above normal under various inflammatory conditions, but most notably if caused by bacterial infections [7]. Therefore, CRP and PCT may be promising markers for rapid detection of infectious complications during SE in the intensive care unit (ICU).

However, SE itself may lead to systemic inflammatory reaction with an increase of cytokines in serum during or immediately after epileptic seizures [8]. Therefore, epileptic activity may also lead to an increase of CRP, PCT and white blood cells (WBC) without the presence of infections and thus reduce the reliability of these biomarkers for the clinical diagnosis of infectious complications during SE.

The aim of this study was to examine whether levels of serum CRP, PCT and WBC are reliable indicators for the diagnosis of infectious complications during SE.

## Material and methods

### Setting

This study was performed at the University Hospital Basel, an 855-bed tertiary care center of Switzerland with over 30,000 admissions per year. Patients with SE are treated mainly in the ICU, which has 21 beds. The local ethical committee EKBB ("Ethikkommission beider Basel") approved this study in accordance with the standards laid down in the 1964 Declaration of Helsinki and waived the requirement for informed consent (approval reference number 204/10).

### Patients and data collection

Over five consecutive years (1 January 2005 to 31 December 2009), patients hospitalized in the ICU due to SE confirmed by electroecephalogram (EEG) were selected from the digital EEG database. EEG recordings were interpreted by two board certified epileptologists (RS and SR) who reached a consensus diagnosis after reviews. CRP and WBC levels were measured daily during the first three days after SE onset. Values of PCT were included if measured during the first 48 hours of SE.

A comprehensive review of medical records and clinical data was performed by a board certified neurologist (RS) and by an infectious disease specialist (ST-S). Data collection included gender, age, all medical diagnoses, duration and type of SE, length of hospital and ICU stay. These data were collected in order to establish a cohort for ongoing analysis regarding the diagnostic value of biomarkers for infectious complications and their correlation with course and outcome of SE.

### Definitions and treatment of status epilepticus

Status epilepticus was defined according to published recommendations [9], as seizure activity, lasting longer than five minutes or recurrent seizure activity lasting more than five minutes without the patient regaining consciousness. Clinically suspected seizure activity was confirmed by EEG. SE episodes were categorized as convulsive (CSE), subtle (SSE), and nonconvulsive status epilepticus (NCSE). The diagnosis of CSE was made if recurrent convulsions were present without complete recovery between seizures. The diagnoses of CSE and SSE were made, as defined by Treiman *et al*. [10]. For the diagnosis of SSE, seizure activity on EEG had to endure when the associated motor responses were fragmentary or partially absent. For the diagnosis of NCSE, at least one of the three primary criteria and one of the four secondary criteria as defined by Young *et al*. had to be fulfilled [11]. SE duration was defined as the period from the time of diagnosis to the time of cessation of clinical manifestations and first EEG without epileptic activity.

Refractory SE (RSE) was defined as SE without recovery after the application of i.v. benzodiazepines and one further i.v. antiepileptic drug, such as phenytoin, valproic acid, a combination of both or levetiracetam. Treatment of SE was standardized according to the guidelines of the Swiss Status Epilepticus Consensus Conference from 2005 [12,13].

### Definitions/criteria of infections

A protocol for monitoring infections was established for all patients on the ICU. It included drawing of blood cultures, urine cultures, cultures from tracheal aspirates or sputum, and performance of a chest x-ray in any patient with new onset of fever or hypothermia. If there were other foci suspected to be the origin of an infection, these were sampled accordingly (for example, cerebrospinal fluid (CSF) cultures, swabs of wounds, cultures of biopsies).

All infections that occurred and were diagnosed 14 days before or during SE were assessed. Patients were categorized into three groups: (1) patients with infections before SE, (2) patients with infections during SE, and (3) patients without infections. Therefore, clinical courses and primary sites of infection were evaluated according to information supplied by the laboratory, clinical and radiological results in the medical records (that is, body temperature, WBC levels, blood cultures, urine cultures, cultures from CSF, cultures from transtracheal aspirates or sputum, antibody titer levels, histopathological results and reports from radiological findings) and from written statements from the primary caring physicians (that is, all written statements relating to clinical signs of possible infections). Furthermore, all microbiological data were obtained during hospitalization and retrospectively cross-checked by a board certified infectious disease specialist (ST-S) by using the computerized database of the infection control microbiology surveillance. Only infections that met the criteria according to the Center for Disease Control (CDC) in the retrospective survey were accepted as infections [14] (Table 1). As the sensitivity of clinical suspicion of VAP is often too high, we applied the criteria of the American Thoracic Society and the Infectious Diseases Society of America [15].

**Table 1:** Identified infections and the applied CDC definitions according to Garner *et al*. [14]

Respiratory tract infections (n = 47; 29.4% of all patients)	RTI must meet one of the following criteria:
	1. Rales or dullness to percussion on physical examination of chest AND any of the following:
	a. New onset of purulent sputum or change in character of sputum b. Organism isolated from blood culture c. Isolation of pathogen from specimen obtained by transtracheal aspirate, bronchial brushing, or biopsy
	2. Chest radiographic examination shows new or progressive infiltrate, consolidation, cavitation, or pleural effusion AND any of the following:
	a. New onset of purulent sputum or change in character of sputum b. Organism isolated from blood culture c. Isolation of pathogen from specimen obtained by transtracheal aspirate, bronchial brushing, or biopsy d. Isolation of virus or detection of viral antigen in respiratory secretions e. Diagnostic single antibody titer (IgM) or fourfold increase in paired serum samples (IgG) for pathogen f. Histopathologic evidence of pneumonia
**Bloodstream infections** (includes laboratory-confirmed bloodstream infection and clinical sepsis) (n = 12; 7.5% of all patients)	*In our study we did not differ between primary and secondary bloodstream infections*
	**Primary bloodstream infections:** Laboratory-confirmed bloodstream infection must meet one of the following criteria:
	1. Recognized pathogen isolated from blood culture AND pathogen is not related to infection at another site
	2. One of the following: fever (>38°C), chills, or hypotension AND any of the following:
	a. Common skin contaminant isolated from two blood cultures drawn on separate occasions AND organism is not related to infection at another site; b. Common skin contaminant isolated from blood culture from patient with intravascular access device AND physician institutes appropriate antimicrobial therapy; c. Positive antigen test on blood AND organism is not related to infection at another site
	**Secondary bloodstream infections:** When an organism isolated from blood culture is compatible with a related nosocomial infection at another site, the bloodstream infection is classified as a secondary bloodstream infection. Exceptions to this are intravascular device-associated bloodstream infections, all of which are classified as primary even if localized signs of infection are present at the access site.

**Symptomatic urinary tract infection** (n = 6; 3.8% of all patients)	**Symptomatic urinary tract infection must meet one of the following criteria:**
	1. One of the following: fever (>38°C), urgency, frequency, dysuria, or suprapubic tenderness AND a urine culture of > = 10^5 colonies/ml urine with no more than two species of organisms
	2. Two of the following: fever (>38°C), urgency, frequency, dysuria, or suprapubic tenderness AND any of the following:
	a. Dipstick test positive for leukocyte esterase and/or nitrate b. Pyuria (310 white blood cells [WBC]/ml^3 or WBC/high-power field of unspun urine) c. Organisms seen on Gram stain of unspun urine d. Two urine cultures with repeated isolation of the same uropathogen with > = 10^2 colonies/ml urine in nonvoided specimens e. Urine culture with < = 10^5 colonies/ml urine of single uropathogen in patient being treated with appropriate antimicrobial therapy f. Physician's diagnosis g. Physician institutes appropriate antimicrobial therapy

**Encephalitis** (n = 5; 3.1% of all patients)	**Encephalitis must meet one of the criteria:**
	1. Organism isolated from culture of brain tissue or dura
	2. Two of the following with no other recognized cause: headache, dizziness, fever (>38°C), localizing neurologic signs, changing level of consciousness, or confusion, AND physician institutes appropriate antimicrobial therapy if diagnosis is made ante mortern AND any of the following:
	a. Organism seen on microscopic examination of brain or abscess tissue obtained by needle aspiration or by biopsy during surgery or autopsy b. Positive antigen test on blood or urine c. Radiographic evidence of infection d. Diagnostic single antibody titer (IgM) or fourfold increase in paired serum samples (IgG) for pathogen

**Meningitis** (n = 1; 0.6% of all patients)	**Meningitis must meet one of the following criteria:**
	1. Organism isolated from culture of cerebrospinal fluid (CSF)
	2. One of the following with no other recognized cause: fever (>38°C), headache, stiff neck, meningeal signs, cranial nerve signs, or irritability, AND physician institutes appropriate antimicrobial therapy if diagnosis is made ante mortem AND any of the following:
	a. Increased white cells, elevated protein, and/or decreased glucose in CSF b. Organisms seen on Gram stain of CSF c. Organism isolated from blood culture d. Positive antigen test on CSF, blood, or urine e. Diagnostic single antibody titer (IgM) or fourfold increase in paired serum samples (IgG) for pathogen

Patients with infections diagnosed more than 14 days before SE onset were excluded from further analyzes of sensitivity and specificity of CRP and WBC for detection of infections during SE.

### Measurement of CRP and PCT

CRP concentrations were determined by an enzyme immunoassay with a detection limit of 0.5 mg/L (EMIT; Merck Diagnostica, Zürich, Switzerland). PCT was measured with a high sensitive time-resolved amplified cryptate emission (TRACE) technology assay (PCT Kryptor^®^, B.R.A.H.M.S. AG, Hennigsdorf, Germany). The assay has a detection limit of 0.02 μg/L and functional assay sensitivity of 0.06 μg/L, that is, 3- to 10-fold above normal mean values. Analyses for different cut-off values, including the threshold of 0.1 ug/L, as recommended by the manufacturer, were performed.

### Statistical analysis

Descriptive analyses for sociodemographic factors, biomarkers of infection, comorbidities and outcomes were performed. Categorical variables were summarized as counts and proportions and continuous variables as means and standard deviations. Logistic regressions were used to determine the associations between the categories "infections" and "respiratory tract infections" (RTI), as the largest subgroups of infections, and the different comorbidities. To analyze the diagnostic accuracy of CRP and WBC levels for detection of infections during SE, all patients with infections before SE and without infections were excluded. When comparing means for two different groups, the Mann-Whitney U-test was used. When comparing more than two groups the Kruksal-Wallis test was applied. Sensitivity and specificity, as well as positive and negative predictive values, were calculated for CRP and WBC, for each of the first three days after SE onset, respectively, using the recommended cut-offs as described above. CRP levels ≤10 mg/L were considered normal, levels greater than 10 mg/L were interpreted as elevated according to the manufacturer's recommendations. Cut-off levels of 10.0 × 10^9^/L were used for WBC. To have an indicator of the change of the different biomarkers with time, this change was calculated for each patient first. This was done by means of a linear regression on the values of CRP and WBC for the three different days of measurements (a linear relationship of CRP and WBC levels with time was assumed). Then logistic regressions or general linear models were used to determine the relationship between the presence of infections during SE and the CRP's and WBC's change with time. To find cut-off points on the level of PCT, which would indicate an infection, receiver operating characteristic (ROC) curves were used and sensitivity and specificity for different cut-off points were calculated. Statistical analysis was performed with SAS version 9.2 (SAS Institute 2002, Cary, NC, USA).

## Results

**Figure 1:**
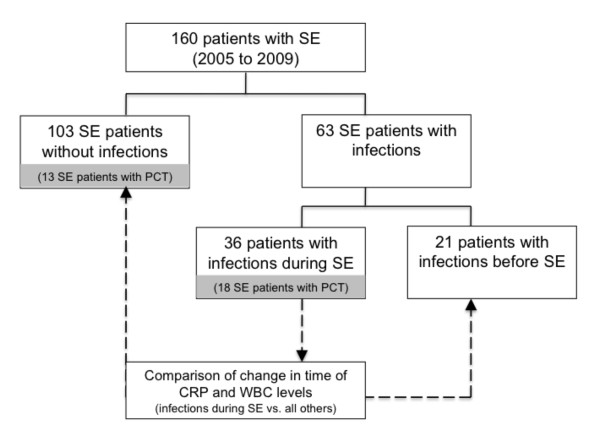
**Flow-chart**. SE = status epilepticus; PCT = procalcitonin.

A total of 160 consecutive patients with SE were identified during five consecutive years in the University Hospital of Basel. The algorithm of patients' in- and exclusion is presented in the flow-chart (Figure 1).

Median age was 65 years (range 17 to 91 years) (Table 2) and cardiopathy and metabolic disorders were the most commonly encountered underlying diseases (55, 34.4% and 46, 28.7%, respectively). Only 29 (18.1%) patients were diagnosed with epilepsy prior to the current episode of SE. The majority of patients suffered from NCSE (72, 45.0%) and nearly one-third had a refractory course of SE (52, 32.5%). Median length of SE was three days (SD ± 5.3). Forty-one (25.6%) patients died. Further baseline characteristics and clinical features are summarized in Table 2. In total, 57 (35.6%) patients had infections before or during SE. Table 1 shows all diagnosed infections and the applied CDC criteria. Thirty-six (22.5%) infections were diagnosed during and 21 (13%) before SE. The majority of infections diagnosed during SE (34/57; 59.7% of all infections) were respiratory tract infections (RTI), more than a third of all RTIs were VAPs (12/34; 35.3% of all RTI during SE and 13.6% of ventilated patients). Relevant pathogens could be detected in most cases of RTI (28/34; 82.4%), and included *Staphylococcus aureus, Streptococci *and gram-negativ bacteria as *Haemophilus spp*., *Escherichia coli*, *Serratia spp*. *Enterobacter spp*. and *Pseudomonas aeruginosa*. In the remaining, diagnosis was established due to clinical findings as new onset of purulent sputum and new or progressive infiltrates in the chest x-ray according to the CDC definitions for nosocomial infections [14] and the criteria of the American Thoracic Society and the Infectious Diseases Society of America [15].

**Table 2:** Basic demographics and clinical features (n = 160)

Sociodemographic			
**Gender**		**n**	**%**
Female		88	55.0
Male		72	45.0
**Age**		**years**	**SD or range**
Mean		62.8	±16.9
Median		65.0	17 - 91

**Clinical features**			

**SE types**		**n**	**%**
CSE		42	26.3
SSE		46	28.7
NCSE		72	45.0
**Comorbidities**		**n**	**%**
Cardiopathy		55	34.4
Metabolic disorder		46	28.8
Known epilepsy		29	18.1
Tumor (in the CNS)		20	12.5
Tumor (outside the CNS)		17	10.6
Neurodegenerative disease		11	6.9
Acute stroke		9	5.6
Traumatic brain injury		8	5.0
Autoimmune disease		8	5.0
Intracerebral hemorrhage		5	3.1
Subdural hemorrhage		4	2.5
Subarachnoideal hemorrhage		3	1.9
Arteriovenous malformation		2	1.3
**Infections of all SE patients**		**n**	**%**
Patients with infections in total		57	35.6
Patients with infections before SE		21	13.1
Patients with infections during SE		36	22.5
**Course**		**days**	**SD or range**
Length of SE	mean	3.0	±5.3
	median	1.0	0 - 43
Length of ICU stay	mean	7.9	±9.1
	median	4.0	0 - 57
Length of hospital stay	mean	20.5	±16.7
	median	17.0	1 - 102
Length of intubation	mean	7.6	±10.1
	median	4.0	0 - 65
Cardiopulmonary resuscitation	n, %	25	15.6
RSE	n, %	52	32.5
**Outcome**		**n**	**%**
Back home		29	18.1
Rehabilitation		47	29.4
Other hospital		32	20.0
Nursing home		10	6.3
Death		41	25.6
Missing data		1	0.6

SD = standard deviation; SE = status epilepticus; CSE = convulsive status epilepticus; SSE = subtle

status epilepticus; NCSE = nonconvulsive status epilepticus; RSE = refractory status epilepticus;

CPR = cardiopulmonary resuscitation; CNS = central nervous system; CRP = C-reactive protein; WBC

= white blood cells; ICU = intensive care unit

To analyze the diagnostic accuracy of CRP and WBC levels for detection of infections during SE, 21 patients with infections before SE were excluded, as already elevated CRP and WBC levels due to prior infection can reduce prognostic value. A total of 139 patients remained for further analyses, of which 36 (27%) had infections during SE. Sensitivity and specificity, as well as positive and negative predictive values of WBC, were low (Table 3). Sensitivity and the negative predictive value of CRP steadily increased from day 1 to day 3 of SE ranging from 70.0% to 93.3% and 85.9% to 96.9%, respectively; however, specificity and positive predictive value remained low (28.9% to 60.2%) (Table 2).

**Table 3:** Validation of C-reactive protein levels and white blood cell counts for infectious complications during SE

**CRP levels** **(mg/L)**		**Infections** **during SE (n = 36)**		**No infections(n = 103)**		**Sensitivity**	**Specificity**	**PPV**	**NPV**	**LR+**	**LR-**
			**SD or range**		**SD or range**						
CRP on day 1 of SE	mean	40.80	44.54	32.35	50.09	70.0%	53.4%	30.4%	85.9%	1.5	1.8
	median	28.35	0.6 - 178.8	7.60	0.3 - 225.5						
CRP on day 2 of SE	mean	77.17	70.13	42.90	53.54	86.7%	37.9%	28.9%	90.7%	1.4	0.4
	median	55.20	0.6 - 245.0	22.00	0.5 - 233.5						
CRP on day 3 of SE	mean	116.04	98.01	62.39	67.99	93.3%	60.2%	40.6%	96.9%	2.3	0.1
	median	92.35	1.0 - 383.8	36.95	1.2 - 256.7						
**White blood cell levels** **(x10^9^/L)**		**Infections** **during SE (n = 36)**		**No infections** **(n = 103)**		**Sensitivity**	**Specificity**	**PPV**	**NPV**	**LR+**	**LR-**
			**SD or range**		**SD or range**						
WBC on day 1 of SE	mean	12.23	6.44	10.71	5.05	72.5%	51.5%	36.7%	82.8%	1.5	0.5
	median	10.50	2.8 - 26.4	10.00	1.2 - 32.7						
WBC on day 2 of SE	mean	9.75	5.09	10.62	5.68	36.7%	55.3%	19.3%	75.0%	0.8	1.1
	median	9.45	1.6 - 29.7	9.80	1.0 - 34.4						
WBC on day 3 of SE	mean	9.52	5.00	9.64	4.64	33.3%	63.1%	20.8%	76.5%	0.9	1.1
	median	8.85	3.0 - 26.0	8.40	0.8 - 23.3						
**Association with infections during SE (Infections during SE vs. no infections during SE) (n = 160)**											
**Marker**	**Linear change with time**					OR*	**95% CI**	**p-value**			
CRP over time	Change from day 1 to 3 (mg/L)					1.018	1.007-1.029	0.0012			
											
WBC over time	Change from day 1 to 3 (x10^9^/L)					0.754	0.603-0.944	0.0137			

SD = standard deviation; SE = status epilepticus; CPR = cardiopulmonary resuscitation; CNS = central nervous system; CRP = C-reactive protein; WBC = white blood cells; PPV = positive predictive value; NPV = negative predictive value; LR+ = likelihood ratio positive; LR- = likelihood ratio negative

Analysis of the linear change with time from day 1 to day 3 for CRP and WBC levels and its association with infections during SE was performed for all 160 patients (comparing results for patients with infections during SE and without infections) and was found to be highly significant for both markers (Table 3). For each 1 mg/L of CRP the linear time change from the first day to the third day after onset of SE was significantly associated with the presence of infections during SE (OR 1.018, CI 1.007 to 1.029, *P *= 0.0012). The change of WBC counts, expressed in 10^9^/L, in the same time period was also associated with the presence of infections during SE (OR 0.754, CI 0.603 to 0.944, *P *= 0.0137).

**Figure 2:**
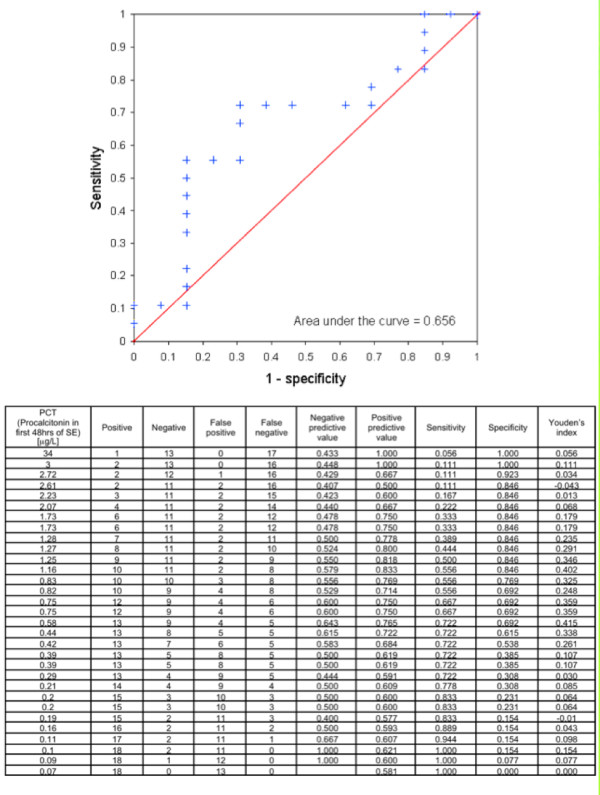
**Receiver Operating Characteristic for procalcitonin levels and development of infections before or during status epilepticus**. PCT = procalcitonin; SE = status epilepticus.

PCT was measured in 31 (19.4%) patients during the first 48 hours of SE. Eighteen of these patients had infections during SE. In all 13 patients without infections, PCT levels were higher than 0.1 μg/L, the recommended threshold value set by the manufacturer (Table 2). Mean PCT level in the entire study group was 2 μg/L (± 6). No significant differences could be detected between PCT levels of patients with infections during SE (mean level 2.92 μg/L ± 7.8) and without infections (mean level 0.73 μg/L ± 0.89) (*P *= 0.15). Regarding the recommended cut-off value of 0.1 μg/L, sensitivity of PCT to detect infections during SE was high (94.4%) with a negative predictive value of 66.7%. However, specificity was low (15.4%) with a positive predictive value of 60.7%. As there are studies that have used higher cut-off values between 0.25 and 0.5 ug/L to withhold antibiotics in patients, that is, considered not to have an infection [16,17], different cut-off values for PCT were analyzed in addition without further improvement of sensitivity and specificity (Figure 2). At the maximum, Youden's index (0.4145) sensitivity was 72.2% and specificity 69.2% at a cut-off of 0.58 μg/L (Figure 2). Baseline characteristics, including age, gender and number of comorbidities, did not differ significantly between the 13 patients with infections during SE and the 18 patients without infections.

## Discussion

To our knowledge, this is the first study to examine the reliability of CRP, WBC and PCT for the diagnosis of infections in patients with SE. Of note, the knowledge about systemic inflammatory reactions with elevated cytokine serum levels during SE is increasing [18-22], accelerating the uncertainty for the clinicians about the use of acute phase proteins for the diagnosis of infections in SE. Numerous studies have identified different cut-off levels for CRP, PCT and WBC for the presence of infections in ICU patients but a consensus was not found [6,23-29].

In our study, CRP demonstrated a high negative predictive value for infections in patients with SE. In addition, its sensitivity steadily increased from Day 1 to Day 3 of SE. However, specificity and positive predictive value were low, making it a poor predictor for the presence of infections during SE. However, the cut-off chosen for CRP (<10 mg/L) is low and might have led to high sensitivity and low specificity. Single values of WBC counts were insufficient to either rule in or rule out the presence of infections during SE. ROC curves were not performed, as linear changes of CRP and WBC levels over time were highly significant with the presence of infections and, as mentioned above, many previous studies have found numerous cut-off values by focusing on infection in ICU patients, making it complex and obscure for the clinicians to rely on multiple recommendations. Therefore, we have focused on the change of biomarker levels over time. In our study population, the linear changes with time for both CRP and WBC levels were significantly associated with the diagnosis of infections, suggesting that both parameters are useful when evaluating their time course rather than single values. For the presented odds ratios, it has to be taken into account that they are given for each change of 1 mg/L of CRP and 10^9^/L for WBC, respectively. This finding might be reflected by the increasing specificity on day 3 of SE in comparison to day 1.

The diagnostic accuracy of PCT could only be analyzed in a small proportion of our patients and only as single measurements. Data for analysis of linear change with time were insufficient. Regarding the recommended cut-off value of 0.1 μg/L, sensitivity of PCT to detect infections was high; however, specificity was low. We conducted further statistical analysis to determine if different cut-off values could improve its performance, however, without relevant success. In a recent study, the diagnostic value of PCT for diagnosis of nosocomial pneumonia has also been questioned [25]. When examining 104 consecutive ICU patients, diagnostic sensitivity and specificity for nosocomial pneumonia was 50.0% and 48.6%, respectively. Of interest, performance of PCT regarding sensitivity was much better in our study at the maximum Youden's index of 0.4145 with a sensitivity of 72.2% and specificity of 69.2%, although our sample size is smaller. In a recent study of 70 patients, PCT analysis for suspected nosocomial infections in ICU patients revealed similar results with a cut-off value for PCT of 0.44 μg/L and a sensitivity of 65.2% and a specificity of 83.0% [24]. The authors also reported poor predictive value of CRP and WBC for detection of hospital-acquired infections. As mean levels of CRP, WBC and PCT were elevated in patients with and without infections during SE and no significant differences in the PCT levels, which is regarded as the most sensitive biomarker for detection of bacterial infections, could be detected in this study, our data suggest that epileptic activity itself may raise serum PCT, CRP and WBC levels. This finding underlines the hypothesis that SE itself could cause a systemic inflammatory reaction - an assumption also reported in the literature by detection of significantly higher levels of cytokines, especially interleukin-6, in the blood of patients with SE [8]. Cytokines are known to influence acute phase protein production, with interleukin-6 being a major inducer [30]. The role of inflammation in maintaining an epileptic state needs further investigation as it could imply new treatment options for this severe and often fatal disease.

A further explanation for elevated biomarker levels without evidence of infections during SE might be the presence of comorbidities in this cohort. Patients with cardiopulmonary resuscitation and cerebral hypoxia have shown PCT elevation in the absence of infections [31] and also multiple trauma with or without brain injury, as present in our study, were shown to be associated with an increase of PCT concentrations without infectious complications [32]. However, there were no significant differences of comorbidities between patients with and without infections and also no differences between patients with and without RTI, the largest subgroup of infections in this study (Table 4). We did not perform analyzes for the subgroup with autoimmune diseases, as this group had a small size of only 5% of the cohort. Moreover, we cannot exclude that there were some cases where infections were present but could not be detected.

**Table 4:** Distribution of comorbidities of patients with and without infections or RTI

For patients with and without infections			
**Comorbidities**	**Odds ratio (unadjusted)**	**95% CI**	**p-Value**

Traumatic brain injury	3.094	0.731 -13.085	0.1248
Cardiopathy	0.747	0.324 -1.723	0.4944
Known epilepsy	0.67	0.231 -1.939	0.4595
Metabolic disease	2.095	0.931 -4.713	0.0739
Stroke	2.25	0.479 -10.579	0.3045
Tumor (in the CNS)	0.46	0.126 -1.673	0.2384
Tumor (outside the CNS)	2.089	0.687 -6.350	0.1941
Neurodegenerative disease	0.299	0.036 -2.442	0.2596

**For patients with and without RTI**			

**Comorbidities**	**Odds ratio (unadjusted)**	**95% CI**	**p-Value**

Traumatic brain injury	1.473	0.337 -6.429	0.6066
Cardiopathy	0.979	0.478 -2.006	0.9545
Known epilepsy	0.724	0.286 -1.832	0.4951
Metabolic disease	1.884	0.911 -3.898	0.0875
Stroke	2.01	0.515 -7.842	0.315
Tumor (in the CNS)	0.385	0.107 -1.382	0.1433
Tumor (outside the CNS)	1.803	0.642 -5.063	0.2633
Neurodegenerative disease	0.224	0.028 -1.801	0.1595

RTI = respiratory tract infection; CNS = central nervous system

The authors are well aware of limitations of this study: the retrospective character and the small patient sample. Nevertheless, as CRP and WBC are measured routinely and daily in every patient on the ICU in clinical practice, a selection bias appears very unlikely. Furthermore, the selection of patients in dependence of performed PCT measurements is an obvious selection bias. The lack of a biomarker-specific time course for PCT in this cohort as well as the smaller sample size does not allow a direct head-to-head comparison to the other markers and may also weaken its prognostic strength for infections, which should be analyzed in further prospective studies. Inter-rater agreement calculation was not performed in this study. However, all EEG reports were based on EEG interpretation by two board certified epileptologists and discrepant interpretation was resolved by consensus. In addition, all microbiological data that were obtained during hospitalization were retrospectively, independently cross-checked by a board certified infectious disease specialist using the computerized database of the infection control microbiology surveillance and only if the required definitions by the CDC were fulfilled, was the diagnosis of infection accepted.

## Conclusions

Single levels of CRP and WBC are not reliable for diagnosis of infections during SE, while linear changes of CRP levels and WBC counts over time are significantly correlated with the presence of infections during SE. In addition, normal levels of CRP and PCT rule out hospital-acquired infections in SE patients. Further studies will be needed to elucidate whether changes in PCT levels over time would be an even more accurate approach for diagnosis of infections during SE.

## Key messages

• Single levels of CRP and WBC are not reliable for diagnosis of infections during SE, possibly due to a systemic inflammatory reaction caused by SE itself.

• Linear changes of CRP levels and WBC counts significantly correlate with the presence of infectious complications during SE.

• Low levels of CRP and PCT rule out hospital-acquired infections in SE patients.

## Abbreviations

CDC, Centers for Disease Control and Prevention; CRP, C-reactive protein; CSE, convulsive status epilepticus; CSF, cerebrospinal fluid; EEG, electroencephalography; ICU, intensive care unit; NCSE, nonconvuslive status epilepticus; PCT, procalcitonin; ROC, receiver operating characteristic; RSE, refractory status epilepticus; RTI, respiratory tract infection; SE, status epilepticus; SSE, subtle status epilepticus; TRACE, time-resolved amplified cryptate emission; VAP, ventilator-associated pneumonia; WBC: white blood cells

## Competing interests

The authors declare that they have no competing interests.

## Authors' contributions

RS, ST-S and SR acquired, analyzed and interpreted the data, as well as substantially contributed to conception and design of the manuscript. LG analyzed and interpreted the data and critically revised the manuscript. AFW and SM made substantial contributions to conception and design of the manuscript and were involved in revising it critically for important intellectual content. All authors have given final approval of the version to be published.
